# Sulfur cycling connects microbiomes and biogeochemistry in deep-sea hydrothermal plumes

**DOI:** 10.1038/s41396-023-01421-0

**Published:** 2023-05-13

**Authors:** Zhichao Zhou, Patricia Q. Tran, Alyssa M. Adams, Kristopher Kieft, John A. Breier, Caroline S. Fortunato, Cody S. Sheik, Julie A. Huber, Meng Li, Gregory J. Dick, Karthik Anantharaman

**Affiliations:** 1 Department of Bacteriology, University of Wisconsin–Madison, Madison, WI 53706 USA; 2 Freshwater and Marine Sciences Graduate Program, University of Wisconsin–Madison, Madison, WI 53706 USA; 3 Microbiology Doctoral Training Program, University of Wisconsin–Madison, Madison, WI 53706 USA; 4 School of Earth, Environmental, and Marine Sciences, The University of Texas Rio Grande Valley, Edinburg, TX 78539 USA; 5 Department of Biology, Widener University, Chester, PA 19013 USA; 6 Department of Biology and Large Lakes Observatory, University of Minnesota Duluth, Duluth, MN 55812 USA; 7 Marine Chemistry and Geochemistry, Woods Hole Oceanographic Institution, Woods Hole, MA 02543 USA; 8 Archaeal Biology Center, Institute for Advanced Study, Shenzhen University, Shenzhen, 518060 China; 9 Shenzhen Key Laboratory of Marine Microbiome Engineering, Institute for Advanced Study, Shenzhen University, Shenzhen, 518060 China; 10 Department of Earth and Environmental Sciences, University of Michigan, Ann Arbor, MI 48109 USA; 11 Cooperative Institute for Great Lakes Research, University of Michigan, Ann Arbor, MI 48109 USA

**Keywords:** Biogeochemistry, Microbial ecology, Microbiome, Population genetics

## Abstract

In globally distributed deep-sea hydrothermal vent plumes, microbiomes are shaped by the redox energy landscapes created by reduced hydrothermal vent fluids mixing with oxidized seawater. Plumes can disperse over thousands of kilometers and their characteristics are determined by geochemical sources from vents, e.g., hydrothermal inputs, nutrients, and trace metals. However, the impacts of plume biogeochemistry on the oceans are poorly constrained due to a lack of integrated understanding of microbiomes, population genetics, and geochemistry. Here, we use microbial genomes to understand links between biogeography, evolution, and metabolic connectivity, and elucidate their impacts on biogeochemical cycling in the deep sea. Using data from 36 diverse plume samples from seven ocean basins, we show that sulfur metabolism defines the core microbiome of plumes and drives metabolic connectivity in the microbial community. Sulfur-dominated geochemistry influences energy landscapes and promotes microbial growth, while other energy sources influence local energy landscapes. We further demonstrated the consistency of links among geochemistry, function, and taxonomy. Amongst all microbial metabolisms, sulfur transformations had the highest MW-score, a measure of metabolic connectivity in microbial communities. Additionally, plume microbial populations have low diversity, short migration history, and gene-specific sweep patterns after migrating from background seawater. Selected functions include nutrient uptake, aerobic oxidation, sulfur oxidation for higher energy yields, and stress responses for adaptation. Our findings provide the ecological and evolutionary bases of change in sulfur-driven microbial communities and their population genetics in adaptation to changing geochemical gradients in the oceans.

## Introduction

Hydrothermal vents are abundant and widely distributed across the deep oceans. The mixing of hot hydrothermally-derived fluids rich in reduced elements, compounds, and gasses, with cold seawater forms hydrothermal plumes [1, 2]. Generally, plumes rise up to hundreds of meters from the seafloor and can disperse over hundreds to thousands of kilometers through the pelagic oceans [3]. Surrounding microbes migrate into the plume and thrive on substantial reductants as the energy sources, making plumes “hotspots” of microbial activity and geochemical transformations [1, 2]. Plumes constitute a relatively closed ecosystem that depends on chemical energy-based primary production and is mostly removed from receiving inputs of energy from the outside [4, 5]. Thus, plumes serve as an ideal natural bioreactor to study the processes and links between microbiome and biogeochemistry and the underlying ecological and evolutionary bases of microbial adaptation to contrasting conditions between energy-rich plumes and the energy-starved deep-sea [2].

The most abundant energy substrates for microorganisms in hydrothermal plumes include reduced sulfur compounds, hydrogen, ammonia, methane, and iron [2]. Amongst these, sulfur is a major energy substrate for diverse microorganisms in plumes across the globe [2, 6–8]. Sulfur transformations in plumes are dominated by oxidation of reduced sulfur species, primarily hydrogen sulfide and elemental sulfur. The metabolic pathways include oxidation of sulfide to elemental sulfur (*fcc*, *sqr*), oxidation of sulfur to sulfite (*dsr*, *sor*, and *sdo*), disproportionation of thiosulfate (*phs*) to hydrogen sulfide and sulfite, disproportionation of thiosulfate to elemental sulfur and sulfate (*sox*), thiosulfate oxidation to sulfate (*sox, tst*, and *glpE*), and sulfite oxidation to sulfate (*sat*, *apr*) [7, 9–11]. Complete oxidation of sulfur would involve oxidation of hydrogen sulfide all the way to sulfate. However, recent observations in other ecosystems indicate that individual microbes rarely possess a full set of the complete sulfide/sulfur oxidation pathway [10, 12], instead individual steps are distributed across different community members. This likely suggests that sulfur oxidation is a microbial community-driven process that is dependent on metabolic interactions, and asks for revisiting sulfur metabolism and biogeochemistry based on a holistic perspective of the entire community.

Recent microbiome-based ecological studies have focused on elucidating a genome-centric view of ecology and biogeochemistry [7, 10, 12–15]. This approach has expanded our understanding of microbial diversity associated with specific energy metabolisms, including sulfur transformations in hydrothermal plumes, the deep sea, and beyond [7, 14, 16–19]. However, the dynamics and microdiversity of the plume microbiome, and relevant biogeochemical impacts remain relatively underexplored [20–24]. Understanding how environmental constraints and selection shape the microdiversity and the genetic structure of plume microbial populations after migration from background seawater can provide fundamental insights into adaptation mechanisms. These insights can also inform future predictions of microbial responses to the changing oceans.

Here, we characterized the ecological and evolutionary bases of the assembly of the plume microbiome, and their strategies for sulfur cycling-based energy metabolisms. We studied globally distributed hydrothermal plume datasets to define a core plume microbiome. We followed this up with synthesis of genome-resolved metagenomics, metatranscriptomics, and geochemistry from three hydrothermal vent sites (Guaymas Basin, Mid-Cayman Rise, and Lau Basin) to unravel community structure and functional links to biogeochemistry, metabolic connectivity within plume and deep-sea communities, and microdiversity in abundant microbial populations. We demonstrate that plume microbiomes have a distinctive community composition and function, that is adapted towards energy conservation, metabolic interactions, and stress response.

## Materials and methods

### Sample information and omics sequencing

Hydrothermal plume and surrounding background samples were collected from the corresponding cruises: *R/V New Horizon* in Guaymas Basin, Gulf of California (July 2004), *R/V Atlantis* and *R/V Falkor* in Mid-Cayman Rise, Caribbean Sea (Jan 2012 and June 2013), two consecutive cruises on the *R/V Thomas G Thompson* in Eastern Lau Spreading Center (ELSC), Lau Basin, western Pacific Ocean (May-July 2009), and *R/V Thomas G Thompson* in Axial Seamount, Juan de Fuca Ridge, northeastern Pacific Ocean (Aug 2015). In brief, Guaymas Basin plume and background samples were collected by 10 L CTD-Rosette bottles and N_2_-pressure filtered on board for microbial specimen collection by 0.2 µm pore size, 142 mm polycarbonate membranes [11]. The samples were preserved immediately in RNAlater. Mid-Cayman hydrothermal plume and surrounding background samples were collected by Suspended Particulate Rosette (SUPR) filtration device [25] mounted to the remotely operated vehicle *Jason II*. SUPR collected water in the volume of 10–60 L from different sampling locations, and these samples were in situ filtered for microbial specimens by 0.2 μm pore size SUPOR polyethersulfone membranes and preserved in RNAlater flooded conical vials and frozen at −80 °C. For Lau Basin samples, SUPR collected samples were in situ filtered by SUPOR polyethersulfone membranes with 0.8 μm and 0.2/0.8 μm pore size for geochemical analysis and microbial specimen collection, respectively [26]. Samples were preserved in RNAlater flooded conical vials and frozen at −80 °C. For Axial Seamount samples, both plume and background samples were collected by a Seabird SBE911 CTD and 10 L Niskin bottles [27]. Samples of 3 L were then transferred into cubitainers, filtered through 0.22 μm Sterivex filters, and preserved for downstream analysis [27].

**Fig. 1:**
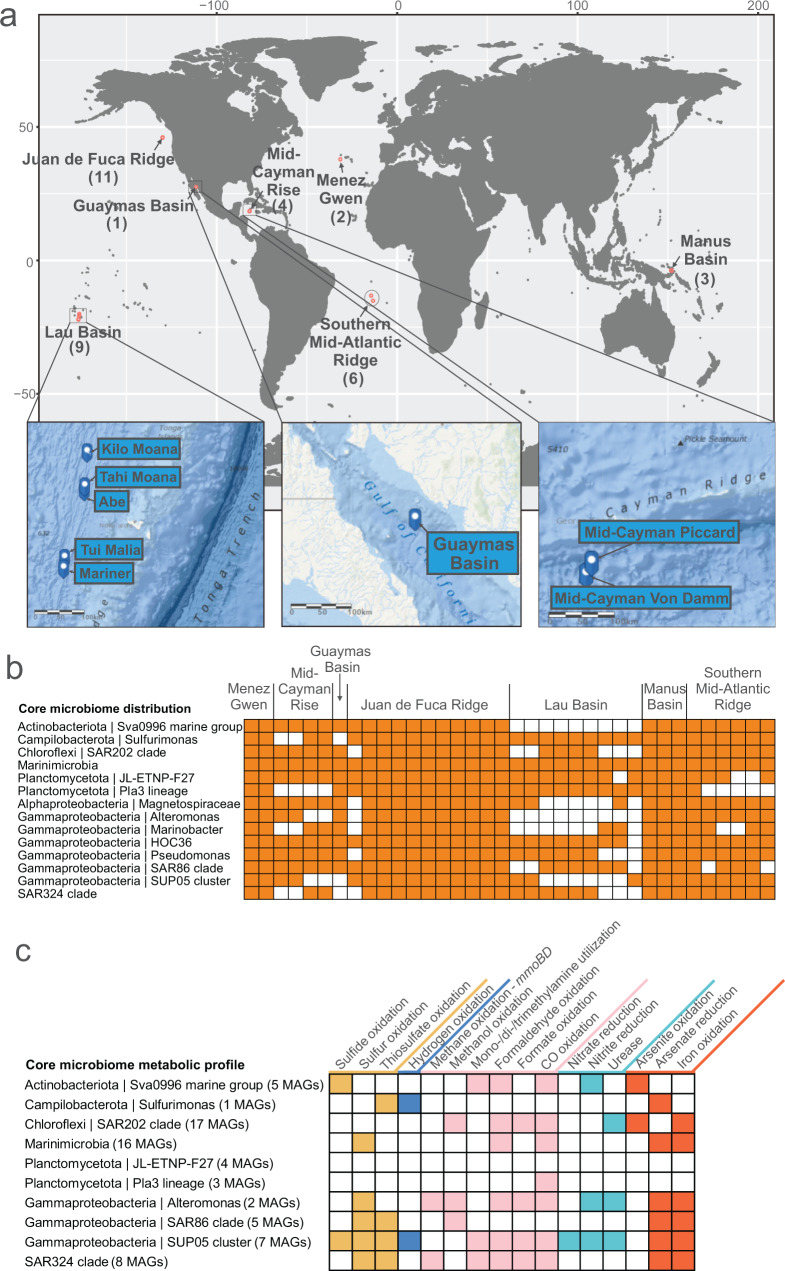
Sampling sites, distribution, and metabolic profile of the core plume microbiome. **a** Sampling site maps of hydrothermal plume samples from which the 16S rRNA gene datasets were sourced. Numbers in brackets indicate dataset quantities. Three hydrothermal sites that have metagenome and metatranscriptome datasets in this study were specifically represented by inset maps. Ocean maps were remodified from ArcGIS online maps (containing layers of “World Ocean Base” and “World Ocean Reference”; https://www.arcgis.com/). **b** Membership and distribution of the core plume microbiome. Heatmap shows the presence/absence of core plume microbial groups (tracing back to known taxonomic ranks from the genus-level taxa) in 36 hydrothermal plume 16S rRNA gene datasets across the world. **c** Metabolic profile of the core plume microbiome. From this study, MAGs that have 16S rRNA genes affiliated to the core plume microbiome were used as representatives (numbers labeled in brackets). This subpanel shows the presence or absence of metabolic potential associated with sulfur, carbon, nitrogen, hydrogen, and metal biogeochemical transformations.

For details of sample collection, preservation, geochemical analysis, and metagenomic/metatranscriptomic sequencing, refer to previous publications [22, 27, 28]. Detailed cruise and sampling information is provided in Supplementary Data 1. The geological map and schematic diagram represent the details of sampling locations (Fig. 1a, Supplementary Fig. S1). The metagenomic DNA and metatranscriptomic cDNA were extracted and synthesized from corresponding samples and processed for HiSeq 2000/2500 (Illumina) sequencing as described previously [11, 14, 18, 27, 29]. The distribution of acquired metagenomes (DNAs, labeled as “D”) and metatranscriptomes (cDNA, labeled as “C”) was represented in Supplementary Fig. S1b (only for samples with detailed location and physicochemical characterization; distribution of other samples refers to Supplementary Data 1). The raw reads (both DNA/cDNA reads) were dereplicated by SeqTools v4.28 (https://www.sanger.ac.uk/tool/seqtools/) and processed by Sickle v1.33 (https://github.com/najoshi/sickle) to trim reads of low quality with default settings. Command “reformat.sh” in BBTools (last modified on Feb 11, 2019; https://www.sourceforge.net/projects/bbmap/) was used to calculate fastq sequence and nucleotide numbers.

### Core hydrothermal plume microbiome analysis

In total, 47 hydrothermal plume and background 16S rRNA gene datasets were used for analyzing the microbiome of hydrothermal plumes, within which 19 datasets were obtained in this study, containing datasets from samples of Mid-Cayman Rise, Guaymas Basin, Lau Basin (Supplementary Data 2). For hydrothermal plume and background samples with only metagenome datasets, 16S rRNA gene sequences were parsed out from metagenomes and these sequences were weighted according to their coverages. Simulated 16S rRNA gene datasets were used in subsequent analyses. The original datasets of paired-end reads were merged into combined 16S rRNA gene tags by FLASH v1.2.11 [30] with default settings. The bioinformatic analyses, including pre-analysis quality control, 16S rRNA gene chimera checking, open-reference OTU picking, taxonomy assignment, OTU table file ‘biom’ generation and rarefaction, OTU representative sequence filtering and alignment, alignment filtering, and phylogenetic tree reconstruction, were performed according to the instructions of QIIME v1.9.1 [31], respectively. The 16S rRNA gene reference database used was “SILVA_138_SSURef_NR99_tax_silva” [32]. The resulting ‘biom’ (OTU table file), ‘tre’ (phylogenetic tree), and “map” (sample characterization map) files were imported into R (using R package ‘*phyloseq*’) for downstream analysis and visualization. Taxa summary and principal coordinates analysis (PCoA) were conducted accordingly to delineate the community structure and biogeographic pattern of hydrothermal plume and background seawater microbiome. Genus-level taxa summary table was used to find the core hydrothermal plume microbiome from 36 hydrothermal plume datasets by filtering genera that exist in >66% plume datasets and have >1% relative abundance on average. Core plume microbiome metabolic profiles were conducted by choosing MAGs (see the following sections for obtaining these MAGs) from this study that contain 16S rRNA genes affiliated to the core plume microbial genera. The approach for metabolic profiling of these MAGs is described in “MAG phylogeny, genomic properties, and protein annotation”.

### Metagenomic assembly and genome binning

QC-processed reads were assembled de novo by MEGAHIT v1.1.2 [33] with settings as “--k-min 45 --k-max 95 --k-step 10”. Hydrothermal plume and background metagenomes from the same hydrothermal site were assembled together. QC-processed reads were re-mapped to assemblies by Bowtie 2 v2.2.8 [34] with default settings. For each hydrothermal site, hydrothermal plume and background reads were mapped to corresponding assemblies separately; bam files by plume and background samples for individual assemblies were used for downstream binning. Subsequently, the assemblies were subjected to a MetaBAT v0.32.4 [35] based binning with 12 combinations of parameters. Afterward, DAS Tool v1.0 [36] was applied to screen MetaBAT MAGs, resulting in high quality and completeness MAGs. This MetaBAT/DAS Tool method enables a comprehensive “slice-layer profiling” for searching potential MAGs with a better outcome (in-house tested). CheckM v1.0.7 [37] was used to assess MAG quality and phylogeny. Outlier scaffolds with abnormal coverage, tetranucleotide signals, and GC pattern within potential high contamination MAGs (by CheckM) and erroneous SSU sequences within MAGs were screened out and decontaminated by RefineM v0.0.20 [38] with default settings. Afterwards, further MAG refinement for decontaminating certain MAGs was conducted by manual inspection based on VizBin [39]. MAGs were picked using a threshold of <10% contamination (namely genome redundancy) and >50% completeness.

### MAG phylogeny, genomic properties, and protein annotation

Genome phylogeny was determined by RefineM and GTDB-Tk v0.2.1 [40] (GTDB database, release 83). Additionally, phylogenies of those genomes that could not be assigned to a meaningful microbial group were inferred from ribosomal protein (RP) trees using the phylogenetic reconstruction method described below. Genomic properties, including genome coverage, genome and 16S rRNA gene taxonomy, tRNAs, genome completeness, and scaffold parameters, were parsed from results that were calculated by CheckM and tRNAscan-SE 2.0 [41]. Relative genome coverages were normalized by setting each metagenomic dataset size as 100 M paired-end reads. MAG ORFs were parsed out by the Prokka annotation pipeline v1.12 [42] with default settings. For ORF annotation, GhostKOALA v2.0 [27], and KAAS v2.1 [26] were applied to thoroughly annotate ORFs to KOs. When combining annotations from different software, we used the resulting KO from the first software as the final annotation; if there was no annotation from the first software, then we moved on to the next software accordingly. Annotation by NCBI nr database (Mar 6, 2017 updated) was conducted with default settings, and for each annotation the first meaningful hit (hit not assigned as ‘hypothetical protein’) was extracted. Genomic-specific metabolic traits were searched against TIGRfam, Pfam, Kofam, and custom HMM profiles using hmmscan [43] and custom protein database using DIAMOND BLASTP [44]. For searching against custom HMM databases, noise cutoff values were determined according to previously reported settings [12]. For DIAMOND BLASTP searches, a stringent criterion of “-e 1e-20 --query-cover 65 --id 65” was applied. Carbohydrate active enzymes (CAZymes) were searched against dbCAN2 with default settings [45]; Peptidases were searched against MEROPS ‘pepunit’ database with stringent DIAMOND BLASTP settings as “-e 1e-10 --subject-cover 80 --id 50” [46].

### Phylogenetic tree reconstruction

The syntenic block of universal 16 ribosomal proteins (RPs) (L2-L6, L14-L16, L18, L22, L24, S3, S8, S10, S17, and S19) were used for inferring RP phylogenetic tree, after hmmscan-based [43] searches for RPs from all MAGs. The individual RP was pre-aligned with local custom RP database by MAFFT v7.123b [47] and curated in Geneious Prime v2019.0.4 [48] by manually masking out the beginning and end regions with lots of gaps. Out of 206 MAGs, 177 containing >4 RPs were used; the concatenated and curated 16RP-alignment (7741 aligned columns) was used for phylogenetic inference by IQTREE-based maximum likelihood method (IQ-TREE multicore v1.6.3 [49]) with settings of “-m MFP -bb 1000 -redo -mset WAG,LG,JTT,Dayhoff -mrate E,I,G,I+G -mfreq FU -wbtl”. The resulting phylogenetic tree was rooted by archaeal lineages and visualized by iTOL [50]. Functional traits were added accordingly to each MAG on the tree. Bacterial and archaeal SSU sequences (>300 bp and the longest from individual MAG) were parsed out (using CheckM ssuFinder [37] to pick and RefineM to filter erroneous hits) and were aligned using SINA aligner [51] with default settings. The 16S rRNA gene taxonomy was checked by BLASTn searches against the “SILVA_138_SSURef_NR99_tax_silva” database [32] and 16S rRNA gene sequences with resulting taxonomy different from their MAG phylogeny (at the phylum level) were filtered due to the high possibility of contamination. IQTREE-based [49] phylogenetic inference was conducted with settings of “-st DNA -m MFP -bb 1000 -alrt 1000”. The 16S rRNA gene tree based on the alignment of 85 sequences with 50000 columns was rooted by archaeal lineages, visualized by iTOL [50], and manually curated.

### Metagenomic and metatranscriptomic read mapping

QC-passed metagenomic reads were mapped to MAGs separately (metagenomic datasets from Guaymas Basin, Mid-Cayman Rise, and Lau Basin sites were mapped individually to the corresponding MAGs) using Bowtie 2 v2.2.8 with default settings [34]. MetaBAT integrated “jgi_summarize_bam_contig_depths” script and homemade Perl scripts were used to calculate MAG coverage (normalized coverage with each metagenomic dataset size set as 100M paired-end reads). QC-passed metatranscriptomic reads (use the same QC-process as described above with an additional SortMeRNA v2.1 [52] rRNA filtering step) were mapped to MAGs separately, with TPM (Transcripts Per Kilobase Million) calculated for individual genes within each genome.

### Statistical comparison of abundances of MAGs and functional traits

Metagenome/metatranscriptome-based MAG mapping results and functional annotations for all the MAGs were summarized individually. Afterwards, significance tests on the differentiation patterns of MAG (also MAG taxonomic group) and functional trait abundances across all the metagenomic/metatranscriptomic samples were calculated by the R package DESeq2 [53]. Log2 Fold Change value with adjusted *p* value (by nbinomWaldTest) < 0.05 was considered as significant. Relative abundances of MAG (also MAG taxonomic group) and functional traits were visualized by R (using R package ‘*pheatmap*’) with the relative abundance being row normalized by removing the mean (centering) and dividing by the standard deviation (scaling). Sunburst figures were generated to depict the relative abundance of MAGs based on metagenomic/metatranscriptomic mapping results, with the significant Log2 Fold Change values labeled to individual MAGs that have differential abundances between different hydrothermal ecological niches (e.g., plume and background).

To find taxa in microbial communities that are responsible for enriched functions (functions that are significantly enriched in each environment), major functions (including functions that are in the categories of carbon fixation, denitrification, sulfur cycling, hydrogen oxidation, methane oxidation, aerobic oxidation, iron oxidation, and manganese oxidation), and specific functions, custom Perl scripts were written to get the corresponding microbial community contribution information (scripts deposited in https://github.com/AnantharamanLab/Hydrothermal_plume_omics_Zhou_et_al._2021). Functional trait results of all MAGs, MAG coverage within the community (all the MAGs included), and targeted function list were used as inputs to conduct the calculation. For environments with metatranscriptomic reads, we also used active MAG coverage (calculated by metatranscriptomic reads mapping result) as the input to calculate microbial community contribution information based on metatranscriptomes.

### Bioenergetic and thermodynamic modeling

Equilibrium thermodynamic reaction path modeling was used to predict chemical concentrations and activity coefficients resulting from the mixing of seawater with end-member vent fluids (Supplementary Table 1). Our thermodynamic modeling builds on the specific plume model implementation described in Breier et al. [54]. The estimated temperature of bottom seawater was sourced according to previous reports [10]. The original chemical data is derived from Reeves et al. [55] and Anantharaman et al. [10]. For each hydrothermal vent system, we choose at least one representative end-member fluid sample(s), respectively (1 for Guaymas Basin, 2 for Mid-Cayman Rise, and 3 for Lau Basin) (Supplementary Table 1).

Bioenergetic and thermodynamic modeling procedures were conducted as described in Anantharaman et al. [7] and Li et al. [18] (For more details refer to Supplementary Information and Tables). Reaction path modeling was performed with REACT, which is a part of the Geochemist’s Workbench package [56]. Conductive cooling was neglected and mixture temperatures were a strict function of conservative end-member fluid mixing. Precipitated minerals were allowed to dissolve and their constituents to re-precipitate based on thermodynamic equilibrium constraints. Thermodynamic data were predicted by SUPCRT95 [57] for the temperature range of 2 °C to end-member vent fluid temperature and a pressure of 500 bar. The estimated biomasses and free energies of individual environments were calculated and their relative abundance changes along the temperature range (2–121 °C) was visualized by R. Two temperatures (3.0 and 4.9 °C) were picked to conduct the biomass and free energy estimation for representing typical plume temperatures in nature.

### Energy contribution and MAG growth rate calculation

Based on metabolic prediction of each MAG, MAG gene coverage, and expression level within each environment, energy contribution for each electron donor was calculated based on gene coverage/expression level and free energy of each catabolic reaction. The contribution ratio of electron donor species was calculated for individual samples respectively. We also included influence of the presence of electron acceptors to energy contribution calculation. To simplify the hydrothermal condition, we only included two major electron acceptors (O_2_ and NO_3_^−^) and used the ratio of these two electron acceptors to infer energy contribution of electron donors at different oxidative conditions.

Microbial genome replication starts directionally from a single origin [58]. Based on metagenomic mapping, at a single time-point the coverage ratio between the replicating origin and terminus of a microbial genome can be used as a proxy to represent the replication rate/growth rate [59, 60]. The growth rate for each MAG was calculated by iRep v1.10 [59] with default settings. MAGs that are from the same environments were pooled together as the input genomes. Sam files that were generated by metagenomic mapping described above were used as the iRep input. Bar charts that reflect the growth rate and significant difference test result (by *t*-test) of MAG taxonomic groups were generated using R packages ‘*ggplot2*’ and ‘*PairedData*’.

### Network complexity analysis

For each community, a bipartite network was built based on reaction/substrate relationships and the percent energy yields for each reaction. Briefly, the plume chemical reaction table for each reaction was stored; within the table, the substrate and product for a reaction were recorded [61]. Then, for each community, reactions (represented as one set of nodes in the bipartite network) with different percent energy yields were connected with substrates and products in the network (represented as the second set of nodes) via directed edges between both sets of nodes. The energy yields are based on the result from “Bioenergetic and thermodynamic modeling” and are represented on the network as node size proportional to the percent energy yield. These networks were constructed using the Python package ‘*networkx*’ [62] (https://networkx.org/).

The network complexity change as a function of reaction energy yield was calculated by the following steps [63]. For each plume community network, the complexity of the network’s structure was measured.

A node was taken from the network; as a consequence, the change in complexity (ΔC) before and after the node was taken was calculated accordingly. The ΔC was assigned to that node as a property representing that node’s contribution to the network’s overall complexity. Then this node was placed back and these steps were repeated for each reaction node [63].

In this study, complexity (C) was calculated by estimating the algorithmic complexity. Because algorithmic complexity cannot be directly computed, we used an estimate known as the Block Decomposition Method (BDM) [64]. The perturbation analysis to calculate each node’s complexity contribution (ΔC) is called Minimal Information Loss Selection, MILS [32]; in this study, successive edge deletion was replaced as node deletion which also works with good performance [33]. This method has been used to characterize complex properties of biological networks and is proven to be a good measure among many other algorithms [63, 64]. For all reaction nodes in each community plume reaction network, we conducted this measurement for each reaction node and came up with the scatterplots.

### Community-level metabolic analysis

MAGs and plume metagenomic reads were used to conduct community-level metabolic analysis using METABOLIC-C v4.0 [65] with default settings. For Guaymas Basin, Mid-Cayman Rise, and Lau Basin sites, all MAGs and plume metagenomic reads from each site were used separately. From METABOLIC-C regular MW-score results, a group of metabolic cycling steps that are important in reflecting the plume substrate metabolisms were specifically selected to make functional network diagrams (using R script ‘*draw_functional_network.R’* from METABOLIC-C). For each site, MW-score table and functional network diagram (based on both all and selected metabolic steps) were generated, respectively.

### Evolutionary analyses

Metagenomic reads from mesopelagic *Tara* Ocean metagenomic datasets (with >800 m depth) [66] were used as the regular ocean environment representatives to compare microdiversity characteristics with that of hydrothermal environments from this study. To simplify analyses, *Tara* Ocean reads from samples collected by filtration with various filter sizes at each station were pooled as one to represent all reads from that station. Both *Tara* Ocean reads and hydrothermal environment reads (including both background and plume environments; background and plume reads were also pooled together individually to simplify analyses and satisfy coverage requirement of each MAG) from this study were first mapped to hydrothermal environment MAGs recovered from individual sites by Bowtie 2 [34] with default settings. After mapping, reads within resulting bam files were filtered according to the following rules to calculate downstream microdiversity parameters: (1) minimum percent identity of read pairs to reference >95%; (2) maximum insert size between two reads <3× median insert size and minimum insert size >50 bp (so only paired reads are retained). Filtering steps were either conducted by inStrain v1.4.1 [67] or inStrain_lite v0.4.0 [68] (for generating bam files) with the same rules. The software inStrain was further employed to calculate microdiversity parameters for each MAG in individual sites from this study. Subsequently, interesting parameters [67] were picked and parsed accordingly from resulting folders, including ‘coverage’ (average coverage depth of all scaffolds of one genome), ‘breadth minCov’ (percentage of bases in the scaffold that have at least ‘min_cov’ coverage), ‘SNV count/(breadth minCov × length)’ (total number of SNVs called on one genome normalized by genome length and breadth minCov), ‘N/S SNV ratio’ (nonsynonymous to synonymous SNV ratio of one genome), ‘r2_mean’ (*R*^2^ mean between linked SNVs), ‘con freq mean’ (mean value of fraction of reads supporting the consensus base within one genome), ‘con freq mean for N SNV’ (mean value of con freq on all nonsynonymous SNV sites), and ‘con freq mean for S SNV’ (mean value of con freq on all synonymous SNV sites). MAGs that have breadth_minCov value < 50% or do not pass the ‘min_cov’ requirement by inStrain were removed from microdiversity analysis in each site.

In order to identify gene-specific selective sweeps in hydrothermal environments, we further pooled reads together into two categories, one contains hydrothermal environment datasets (including both background and plume environment datasets) and the other contains *Tara* Ocean samples (all *Tara* Ocean sample datasets were pooled together). After read mapping and filtering as described above, *F*_*ST*_ (fixation index) between hydrothermal and *Tara* Ocean environments was calculated using scikit-allel package [69] (Hudson method [70]) within inStrain_lite to identify genes with skewed allele frequencies across the whole genome. Subsequently, high *F*_*ST*_ genes from each MAG within each hydrothermal vent site were identified if they have *F*_*ST*_ value > *F*_*ST*_ mean (genome-wide *F*_*ST*_ average) + 2.5 × *F*_*ST*_ std (genome-wide *F*_*ST*_ standard deviation) and the lowest gene coverage in either hydrothermal and *Tara* Ocean environment samples was higher than 5×. Meanwhile, for each genome the threshold for number of genes with empty *F*_ST_ value was specified to not be more than half of all genes, else high *F*_*ST*_ genes were not taken into account for this genome. We set gene coverage in both environments to be at least 5× due to the fact that reduction of gene coverage (or loss of coverage in some genome regions) can also lead to low nucleotide diversity. Furthermore, to confirm that these genes are specifically selected in hydrothermal environment, additional requirements were added: (1) gene nucleotide diversity in hydrothermal environment < nucleotide diversity genome average in hydrothermal environment; (2) gene N/S SNV ratio in hydrothermal environment > N/S SNV ratio genome average in hydrothermal environment; (3) gene nucleotide diversity in hydrothermal environment < gene nucleotide diversity in *Tara* Ocean samples; (4) gene N/S SNV ratio in hydrothermal environment > gene N/S SNV ratio in *Tara* Ocean samples.

To find sulfur metabolizing genes that have signals of being fixed after migration, a relatively less stringent set of criteria were used to screen gene *F*_*ST*_ values compared to the high *F*_*ST*_ gene identification method in the above paragraph. For each sulfur metabolizing gene (including genes of *sat*, *aprA*, *sdo*, oxidative *dsrAB*, and *soxBCY*) containing MAGs, the identified genes needed to meet the following criteria: (1) *F*_*ST*_ value > *F*_*ST*_ mean (genome-wide *F*_*ST*_ average) and both *F*_*ST*_ and *F*_*ST*_ mean should be positive values; (2) gene nucleotide diversity in hydrothermal environment < gene nucleotide diversity in *Tara* Ocean samples; (3) gene N/S SNV ratio in hydrothermal environment > gene N/S SNV ratio in *Tara* Ocean samples; (4) gene coverages in hydrothermal environments and *Tara* Ocean samples both > 5×. Sulfur metabolizing genes that meet all the four criteria were indicated to have positive gene fixation signals though the selective power across the genome did not reach the level of gene-specific selective sweeps as indicated by the above method.

## Results

We used publicly available microbiome data from hydrothermal vent plumes across the globe to (1) define the core plume microbiome, (2) investigate plume microbiome structure, function, and activity, and (3) identify links between plume microbiomes and geochemistry. To investigate the core microbiome, we studied publicly available 16 S rRNA gene datasets of hydrothermal plumes (*n* = 36) and background deep-sea samples (*n* = 11) from seven ocean basins across the globe. To study the microbiome structure, function, and activity, we reconstructed metagenome-assembled genomes (MAGs, *n* = 206) from three hydrothermal vent sites (containing both plume and background samples from Guaymas Basin, Mid-Cayman Rise, and Lau Basin). We also mapped paired metatranscriptomes from the same sites for some samples (Fig. 1, Supplementary Fig. S1, and Supplementary Data 1). To study links between biogeochemistry and the microbiome, we analyzed paired geochemical data from the above three hydrothermal vent sites. To provide clarity on the plume and background samples and DNA/cDNA libraries used in this study, we have provided a schematic diagram describing the locations of all samples in the context of a hydrothermal vent system (Supplementary Fig. S1).

### Defining the core hydrothermal plume microbiome

To identify and study the core hydrothermal plume microbiome, we used 16S rRNA gene datasets from 47 hydrothermal plume and background deep-sea samples spread across seven ocean basins (Supplementary Data 2). Biogeographic patterns were delineated by UniFrac metrics of distance and PCoA-based ordination. Sample location influenced biogeographic patterns more than sample characteristics (plume/background) (Supplementary Figs. S2 and S3). Unweighted UniFrac PCoA plots indicated that paired plume/background deep-sea samples within the same site were closely correlated (Supplementary Fig. S3). As revealed previously [2, 26, 29], this supports the understanding that microorganisms in hydrothermal plumes are primarily derived from surrounding seawater with dispersal limitation having little effects locally.

We then identified genus-level taxa distributed in plumes with high prevalence and relative abundance. The core plume microbiome consists of 14 microbial groups (Fig. 1a, b) (see Materials and methods). Next, we characterized metabolic profiles for the core plume microbiome by selecting MAGs from this study that were affiliated with the same taxa (Fig. 1c). These organisms demonstrated highly versatile metabolic potential for utilizing various plume substrates [2], including HS^-^, S^0^, H_2_, CH_4_, methyl-/C_1_ compounds, arsenite, and iron (Fig. 1c). We discovered that the majority of the members of the core plume microbiome likely originated from seawater, which is consistent with previous reports [26] (Supplementary Table 3). We also observed a small number of vent chimney/seafloor/subsurface dwelling and endosymbiotic microorganisms that might be entrained in plumes [2, 71] (Supplementary Table 3). Collectively, our data suggest that sulfur and other reduced organic/inorganic compounds shape the global core plume microbiome.

### Sulfur-dominated geochemistry influences energy landscapes and promotes microbial growth

**Fig. 2:**
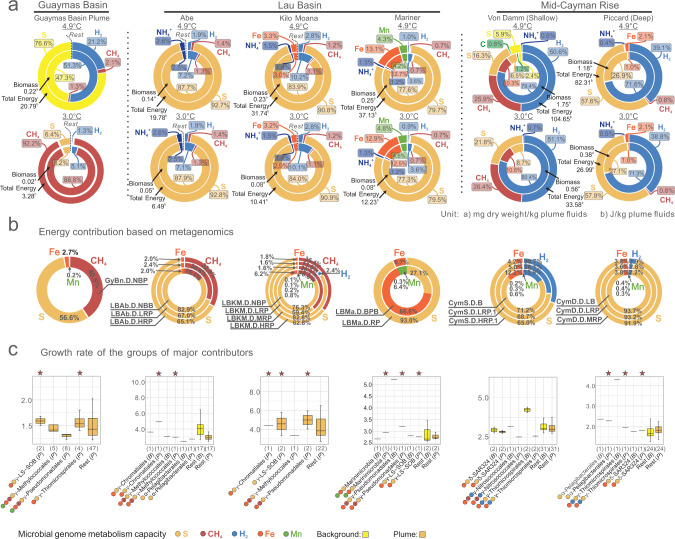
Thermodynamic estimation of available free energies and biomass yields from electron donors, metagenomics-based contribution of electron donors to energy, and growth rates of microorganisms depending on primary energy sources. **a** Thermodynamic estimation diagram of available free energy and biomass. For each hydrothermal environment, the contribution fraction of each electron donor species was labeled accordingly in the rings. The total available free energies and biomass were labeled accordingly to individual plumes. Two temperatures (3.0 °C and 4.9 °C) were picked to represent in situ temperatures in the upper and lower plume. Light yellow represents anaerobic sulfur oxidation, dark yellow represents aerobic sulfur oxidation. Detailed data and estimation diagrams are provided in Fig. S9 and Supplementary Data 8. **b** Metagenomics-based estimation of energy contribution. Energy contribution for each electron donor was calculated based on metagenomic abundance of each reaction of electron donors and free energy yield of each reaction. The contribution ratio of electron donor species was calculated for individual environments respectively. For detailed results refer to Supplementary Data 9. **c** Growth rate of microorganisms depending on main energy sources in each hydrothermal environment. The y-axis for each barplot indicates the replication rate. The microbial groups starting with “α-”, “γ-”, and “δ-” represent Alphaproteobacteria, Gammaproteobacteria, and Deltaproteobacteria, respectively. Plume microbial groups were colored by dark yellow, background microbial groups were colored by light yellow and they were also all labeled with “(*P*)” or “(*B*)”, respectively. Numbers in brackets indicate MAG numbers in each microbial group. Star-labeled plume microbial groups had higher growth rates than the “Rest” plume microbial groups.

Previous thermodynamic modeling analyses have reflected energy landscapes for various hydrothermal ecosystems [4, 7, 10, 16] by representing free energy yields for reactions of various energy sources for microbial metabolism in hydrothermal fluids. These studies have shown that thermodynamic modeling and omics-based biogeochemical estimations are consistent in individual ecosystems [7, 10, 16]. Here based on geochemical parameters and functional profiles of MAGs (Supplementary Figs. S4, S5, S6, and Supplementary Data 3, 4), we conducted an across-site comparison of thermodynamic modeling and omics-based biogeochemical estimations to observe and reflect the influences of distinctive plume geochemical characteristics on plume microorganisms. We also performed growth rate analyses to identify and characterize microbial energy contributors which are favored with faster growth rates in response to distinct plume geochemistries. We first used thermodynamic modeling to reconstruct plume energy landscapes. (Fig. 2a). Guaymas Basin plume energy sources were mainly attributed to sulfur, methane, and hydrogen. Sulfur dominated as the major energy source among Lau Basin plumes, while methane, Mn/Fe, and other energy sources likely play minor roles in microbial metabolism. Finally at Mid-Cayman Rise, two distinct patterns were observed. Plume energy sources at the Von Damm site were hydrogen, methane, and sulfur, while at the Piccard site, plume energy sources were primarily hydrogen and sulfur.

When comparing among sites, distinct geochemical characteristics support the predicted energy landscapes. Specifically, energy sources that are prevalent at high concentrations frequently show high contributions to the energy landscape. Methane was the highest in end-member fluids from Guaymas Basin (63.4 mmol/kg) [7], which supported the dominance of methane oxidation in the Guaymas Basin plume in the thermodynamic model (Fig. 2a); additionally, significant contributions of methane oxidation in metagenomics datasets of Guaymas Basin were also found (~40.5%) (Fig. 2b). Meanwhile, Lau Basin hydrothermal fluids had high Mn and Fe concentrations (Mn: 3.9–6.3 mmol/kg, Fe: 3.8–13.1 mmol/kg) [72, 73] in the Mariner hydrothermal field compared to other samples. This manifested in Fe and Mn oxidation contributing the highest fractions (Mn: ~4–5%, Fe: 13%) in thermodynamic modeling (Fig. 2a) and the highest fractions (Mn: 0.3-6.4%, Fe: 6.7-66.6%) in omics-based energy estimations of Mariner across all sites at Lau Basin (Fig. 2b). Similarly in Mid-Cayman Rise, high hydrogen concentrations in the vent fluids were associated with high contribution of hydrogen oxidation in the thermodynamic model and omics-based estimations (Fig. 2a, b, Supplementary Table 1). Overall, reduced sulfur was the primary energy source in all three sites, as evidenced by thermodynamic modeling and omics-based biogeochemical estimations. However, individual plume geochemical conditions, on the other hand, vary with several diverse minor energy sources such as iron, manganese, methane, and hydrogen, resulting in different energy landscapes mediated by microorganisms.

To study whether microorganisms conducting biogeochemical transformations in each site were also growing actively, we predicted microbial growth rates from metagenomic data using iRep [59]. iRep can calculate the difference in read abundance between the origin and terminus of a genome, which is a proxy for the organism’s replication or growth rate [58–60]. Certain sites showed a consistent pattern that microorganisms depending on main energy sources in plumes such as sulfur have higher predicted growth rates. For instance, members of LS-SOB and *Thiomicrospirales* (previously SUP05 cluster as listed in Fig. 1; *Thiomicrospirales* in GTDB R83 or PS1 in GTDB R202) both had the capacities for sulfur and iron oxidation, and were predicted to have a higher growth rate than other microorganisms in the Guaymas Basin plume (Fig. 2c). Similarly, members of *Methylococcales* and *Chromatiales* had capacities for iron, methane, and sulfur oxidation in Lau Basin (Abe plume) and their growth rates were higher than other organisms (Fig. 2c). Manganese-oxidizing members of *Marinimicrobia* had a higher growth rate than other organisms in the Lau Basin Mariner plume, consistent with thermodynamic modeling-based and omics-based results that Mariner had the highest energy contributions from Mn oxidation among all ecosystems (Fig. 2). Collectively, we discovered a consistent pattern indicating that microorganisms depending on the primary energy sources in plumes have higher predicted growth rates, possibly as a result of their ability to respond to varying geochemistry in hydrothermal plumes.

### Consistency of links among geochemistry, function, and taxonomy

MAGs reconstructed from hydrothermal vents in the Guaymas Basin, Mid-Cayman Rise, and Lau Basin, as well as corresponding omics-based profiling, allowed for taxonomic and functional comparisons across the three sites (Supplementary Figs. S4, S5, S6, and Supplementary Data 3, 4). Across-site analyses of functional traits in MAGs showed that different functions were significantly enriched in different plumes in accordance with the underlying geochemistry, e.g., arsenate reduction and long-chain alkane (C_6_+) degradation in the Lau Basin; CO and methanol oxidation in the Mid-Cayman Rise; and toluene and benzene degradation in the Guaymas Basin (Fig. 1c, Supplementary Fig. S7b). Consistent with the differentially enriched functions, the distribution and abundance of some microbial groups were also significantly enriched in the corresponding samples suggesting linkages between function, distribution, and abundance of microbial groups in plumes (Supplementary Fig. S7a) Examples include arsenate reduction in background deep-sea samples from Lau Basin which was attributed to members of *Bacteroidetes* and *Thiomicrospirales* while the same function in Lau Basin plumes was attributed to only members of *Thiomicrospirales*. CO oxidation in Mid-Cayman plumes was attributed to members of *Chloroflexi*, and toluene and benzene degradation in Guaymas Basin plume were attributed to members of *Methylococcales* and *Pseudomonadales* (Supplementary Data 5). These observations are consistent with hydrothermal vent fluid geochemistry, e.g. Lau Basin hydrothermal vents have high arsenic end-member concentrations [74] (ranging from 2.1 to 11 μmol/kg) and Guaymas Basin fluids contain aromatic hydrocarbons (primarily benzene and toluene) [75].

As for within-site comparisons, the data indicated that the top three contributing taxa for major functions (including eight categories of carbon fixation, denitrification, sulfur cycling, hydrogen oxidation, methane oxidation, aerobic oxidation, iron oxidation, and manganese oxidation) are largely shared between plume and background deep seawater in Mid-Cayman Rise and Lau Basin, indicating functional consistency which was linked to taxonomy (Supplementary Data 5). Nonetheless, the abundance of taxa varied between plumes and the background deep sea (Supplementary Data 5, 6). It is possible that the differences in taxa underlie functional differentiation and they are both triggered by plume geochemical stimulus. For example, members of *Thiomicrospirales* are the major contributors to Rubisco form I-based carbon fixation, oxygen metabolism, nitrate/nitrite reduction, sulfur oxidation, and thiosulfate oxidation based on metatranscriptomic profiling, and these functional traits had higher expression in the Mid-Cayman Rise Von Damm plume compared to the background deep sea. Consistently, members of *Thiomicrospirales* have higher expression levels in Von Damm plume compared to the background (Supplementary Fig. S8b, c, Supplementary Data 6, 7, and more evidence can be found within it). Our results suggest the adaptation of the plume microbiome to its local geochemical environment, and demonstrate the consistency of links between taxonomy, function, and geochemistry.

### Sulfur cycling drives microbial metabolism and metabolic interactions in hydrothermal plumes

**Fig. 3:**
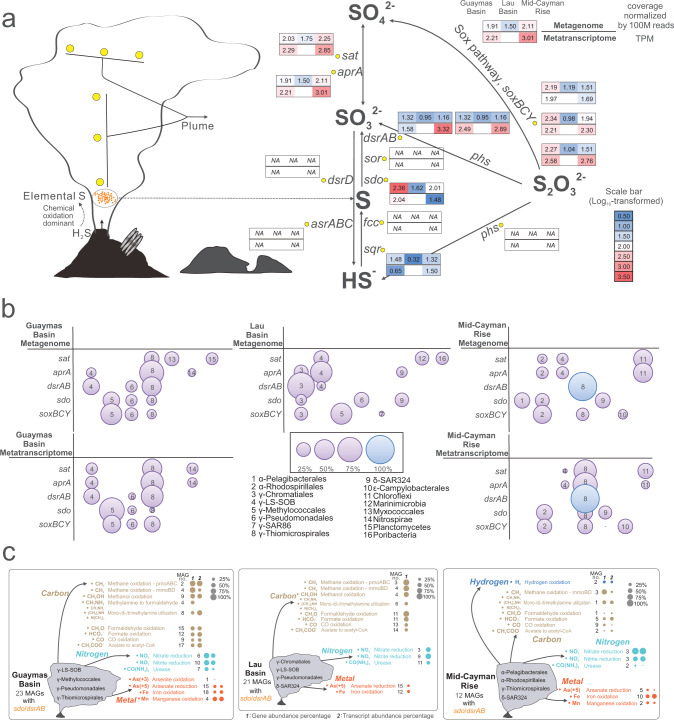
Sulfur metabolism and metabolic plasticity of sulfur oxidizers. **a** Details of sulfur metabolism pathways in the hydrothermal plume. The gene abundance (coverage normalized by 100 M reads) and transcript expression level (TPM) for each step were calculated based on plume metagenomic and metatranscriptomic read mapping results. The metagenomic mapping was conducted separately within individual hydrothermal sites; the metagenomic reads from Guaymas Basin, Mid-Cayman Rise, and Lau Basin sites were mapped individually to the MAGs reconstructed from the corresponding sites. Log_10_-transformed values of gene abundance and transcript expression level were labeled accordingly in the diagram. **b** Major contributors to sulfur metabolizing genes. For each sulfur metabolizing gene, microbial groups that occupied >10% of the total gene abundance (by metagenome) or transcript expression (by metatranscriptome) values were labeled in the diagram. For some genes with only three or less than three contributors, all contributors were labeled. **c** Metabolic plasticity of sulfur oxidizers. For each hydrothermal vent site, three parameters were given to show the metabolic plasticity of sulfur oxidizers in conducting each electron transferring reaction related to carbon, nitrogen, hydrogen, and metal biogeochemical cyclings: the number of sulfur-oxidizing gene containing MAGs, gene abundance percentage, and transcript abundance percentage. The metagenomic/metatranscriptomic mapping was conducted by combining MAGs from each hydrothermal vent site for the analyses described within this figure.

**Fig. 4:**
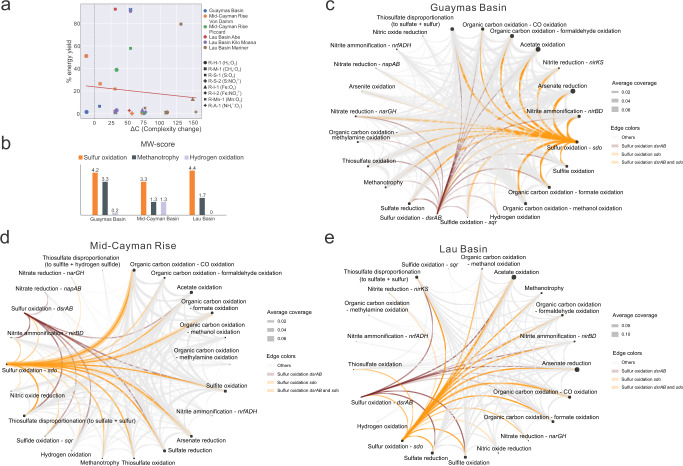
Network complexity, MW-scores (metabolic weight scores), and functional network diagrams of the three hydrothermal vent sites. **a** Network complexity diagram representing each reaction’s influence on the complexity of the network. In the figure, different colors represent different hydrothermal environments, different symbol shapes represent different reactions. The substrates (including electron donors and acceptors) were listed for each reaction in the legend. The x-axis is the change in complexity (ΔC) of the whole network for a node (a reaction here) and the y-axis is the percent energy yield of that reaction in the whole community. This network complexity diagram was based on thermodynamic estimation results at 3.0 °C. **b** MW-scores of three major energy contributing reactions. **c** Functional network diagram of Guaymas Basin. **d** Functional network diagram of Mid-Cayman Rise. **e** Functional network diagram of Lau Basin. A group of metabolic cycling steps that are important in reflecting the plume substrate metabolisms were selected from METABOLIC-C regular MW-score results to make these functional network diagrams (**c**–**e**), respectively. In each functional network diagram, the size of a node is proportional to gene coverage associated with the metabolic/biogeochemical cycling step. The thickness of the edge represents the average gene coverage values of the two connected metabolic/biogeochemical cycling steps. Edges related to two reactions of sulfur oxidation were colored accordingly in each diagram.

Building on our findings from both thermodynamic modeling and omics-based biogeochemical estimations which indicated the importance of sulfur-based metabolisms, we studied microbial metabolic interactions associated with sulfur cycling in all plumes. We recently developed a metric, metabolic weight score (MW-score) [65] to measure the contribution of metabolic/biogeochemical steps, and their metabolic connectivity in a microbial community. More frequently shared functions and their higher abundances in a microbial community lead to higher MW-scores [65]. Both metagenomics and metatranscriptomic data for microbial communities in individual hydrothermal vent sites showed elemental sulfur oxidation to be the key reaction in the sulfur cycle (Fig. 3a). In each community, sulfur oxidation had the highest MW-score (Fig. 4b, Supplementary Fig. S10). Major contributors (*dsrAB* and *sdo* containing MAGs) to sulfur oxidation varied across hydrothermal vent sites (Fig. 3b), indicating that core sulfur oxidizers can have distinct local distributions. Metabolic plasticity was observed in that some sulfur oxidizers had additional metabolic potential associated with utilizing various small carbon substrates and hydrogen, reducing nitrate/nitrite, and oxidizing iron/manganese/arsenite [76] (Fig. 3c). Additionally, numerous connections of sulfur oxidation with other electron-transferring reactions were observed in the functional network (Fig. 4c–e and Supplementary Fig. S10). Previously, sulfur-oxidizing bacteria belonging to *Thiomicrospirales* and SAR324 lineages were identified to have metabolic plasticity involving the ability to conduct hydrogen oxidation and nitrate reduction [7, 77] (in the case of *Thiomicrospirales*) and alkane/methane/carbon monoxide oxidation [17, 78] (in the case of SAR324), implying that plume microorganisms are optimized to mediate energy transformations depending on available electron donors and acceptors. Based on these findings, we posit that sulfur oxidizers are the primary group involved in energy scavenging using plume substrates. Sulfur oxidizers have metabolic plasticity that allows them to connect sulfur metabolism with other elemental transformations, and they contribute significantly to biogeochemical cycles in the deep sea.

While sulfur oxidation connects other metabolic reactions in the overall functional network and has significant energy yields, its role in the overall network complexity, i.e., the impact of sulfur metabolism on overall plume microbial metabolism, remains elusive. To address this, we built networks based on reactions and the percent energy yields, and investigated reaction influence on network complexity [61, 63, 64] (Fig. 4a, Supplementary Fig. S11). The network of reactions works as a whole mechanism [63]. In the network, each reaction is one constitutional part. The high ΔC (complexity change) reactions are key features of the networks. Most of these ΔC values are positive except for two points (Fig. 4a, Supplementary Fig. S11). This indicates that all but two of these reaction nodes drive the system away from randomness and significantly contribute to the complexity of the network as a whole [63]. Meanwhile, in general, it seems that most reactions that are closer to smaller ΔC have higher percent energy yields associated with their reactions (Fig. 4a, Supplementary Fig. S11). This phenomenon suggests that reaction nodes that result in higher changes of percent energy yields are not necessarily contributing to the reaction network’s complexity the most. Overall, our results indicate that, while sulfur oxidation has higher energy yields, other reactions in plumes are also important components that cohesively contribute to the energy landscape.

### Low diversity, short migration history, and gene-specific sweeps in plume populations

**Fig. 5:**
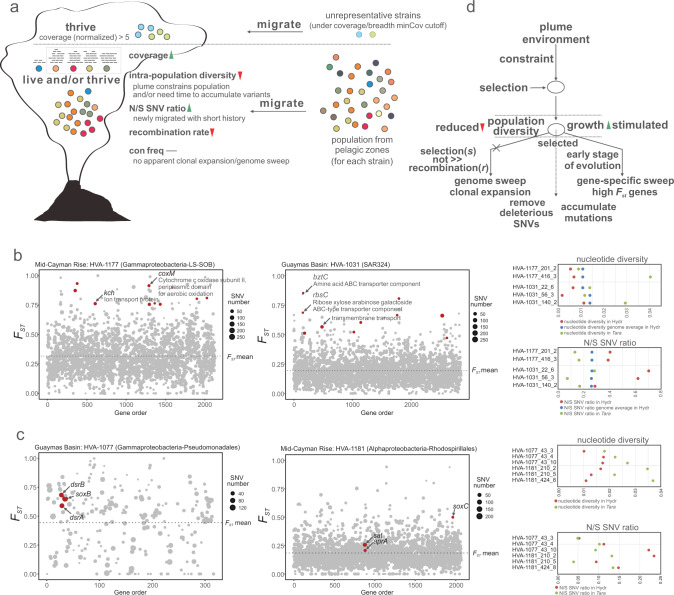
Evolutionary mechanism of plume microbial populations during migration. **a** Schematic diagram showing the changing trend of microdiversity parameters during migration. Individual solid dots with various colors represent microbial populations. Two scenarios were depicted in this panel: unrepresentative strains and strains that have detectable read mapping results in both environments. **b** Two representative charts showing *F*_*ST*_ distribution in MAGs that contain high *F*_*ST*_ genes. In each chart, the x-axis represents gene numbers (only genes with detectable *F*_*ST*_; negative values were removed). Dot sizes were proportional to SNV numbers in individual genes, and *F*_*ST*_ genome-wide mean was depicted in each chart with dashed lines. Red-colored dots represent high *F*_*ST*_ genes that also passed the requirements of *F*_*ST*_, nucleotide diversity, N/S SNV ratios, and coverages (see methods). The nucleotide diversity and N/S SNV ratio distribution for high *F*_*ST*_ genes and genome-wide mean of all genes in different environments were depicted in the chart on the right side. Details of high *F*_*ST*_ genes and related parameters in individual genomes (all hits, also including these two representative genomes) were listed in Supplementary Data 12. **c** Two representative charts showing *F*_*ST*_ distribution in MAGs that contain sulfur metabolizing genes with signals of being fixed. In each chart, the x-axis represents gene numbers (only genes with detectable *F*_*ST*_; negative values were removed). Dot sizes were proportional to SNV numbers in individual genes, and *F*_*ST*_ genome-wide mean was depicted in each chart with dashed lines. Red-colored dots represent sulfur metabolizing genes that passed the requirements of *F*_*ST*_, nucleotide diversity, N/S SNV ratios, and coverages (see methods). The nucleotide diversity and N/S SNV ratio distribution for sulfur metabolizing genes in different environments were depicted in the chart on the right side. Details of sulfur metabolizing genes with signals of being fixed and related parameters in individual genomes (all hits, also including these two representative genomes) were listed in Supplementary Data 13. **d** Diagram showing the underlying evolutionary processes during migration. Circles represent microbial populations. Dash line arrows indicate the direction of the next evolutionary step.

Metagenomes provide full repertoires of genomic variation and facilitate interpreting fine-scale evolutionary mechanisms [67, 79, 80]. Here, we used *Tara* Ocean metagenomic datasets [66] from the mesopelagic oceans to compare metagenomes from hydrothermal plume environments to the wider pelagic oceans and study the population genetic diversity of each MAG (Supplementary Data 10). We discovered that a large proportion of MAGs had a similar tendency in terms of normalized single nucleotide variation (SNV) counts, nonsynonymous/synonymous SNV substitution ratio (N/S SNV), and genome-wide mean *R*^2^ (Fig. 5a and Supplementary Data 11). Hydrothermal plumes have a lower SNV count than *Tara* Ocean samples, a higher N/S SNV ratio, and a higher mean *R*^2^ than *Tara* Ocean samples. This suggests that in the plume: (1) Fewer SNVs are present, and population diversity is lower; (2) The population is younger with a short migration history. The higher N/S SNV ratio indicates that younger populations are less subjected to purifying (negative) selection to remove deleterious mutations; (3) The population is less subjected to recombination. The higher mean *R*^2^ reflects higher SNV linkage frequency at the genome-wide scale, indicating a lower recombination rate among population members.

We also looked into the fine-scale evolutionary parameters to investigate potential signals of genome/gene sweeps. Consensus base frequency (abbreviated as con freq, frequency of reads supporting the consensus base), con freq for nonsynonymous SNV, and con freq for synonymous SNV at the genome-scale level all showed no significant differences (Supplementary Data 11). This indicates that these populations are unlikely to have undergone selective genome sweeps and clonal expansion during migration. We calculated the fixation index *F*_*ST*_ [81] based on gene allele frequencies between these two environments (Fig. 5b and Supplementary Data 12) to investigate environmental selection. High *F*_*ST*_ genes are potential loci where selective pressures act on and they indicate adaptation for microbes after migrating to new niches [68]. Further stringent criteria require lower gene nucleotide diversity and higher N/S SNV ratio (Fig. 5b and Supplementary Data 12). Decreases of nucleotide diversity indicate gene-specific selective sweep in the hydrothermal environment and higher N/S SNV ratios suggest that these genes underwent a recent selection compared to the genome average and their counterpart genes in *Tara* Ocean samples. Amongst 260 identified high *F*_*ST*_ genes using our stringent criteria, many of them involved transporters, aerobic oxidation, and stress responses (Fig. 5b and Supplementary Data 12). Transporters were associated with diverse substrates, e.g., metals (Co, Fe, and Mg), amino acids, Na^+^/H^+^, anions (nitrate/sulfonate/bicarbonate), carbohydrates (ribose/xylose/arabinose/galactoside), and aliphatic polyamines (spermidine/putrescine); meanwhile, these transporters were associated with many transporter families (Supplementary Data 12), including ABC superfamily, tripartite ATP-independent periplasmic (TRAP) family, tripartite tricarboxylate transporter (TTT) family, and others. This suggests that gene-specific selection sweeps have important impacts on nutrient uptake, aerobic oxidation on substrates for higher energy yields, and stress responses.

Given the observed importance of sulfur metabolism in plumes, we focused on the 238 identified sulfur metabolism genes. With *F*_*ST*_ values higher than the genome average, 23 of these genes showed signs of being fixed after migration (Fig. 5c and Supplementary Data 13). These genes were associated with sulfur oxidation, thiosulfate oxidation, and sulfite oxidation/sulfate reduction (*sat*, *aprA*, *sdo*, oxidative *dsrAB*, and *soxBC*) (Supplementary Data 13). This demonstrates that, despite not reaching the level of gene-specific selection sweeps, these sulfur metabolizing genes were still being selected across the genome. Overall, this suggests a genetic adaptation to a sulfur-dominated environment after migration. An underlying evolutionary paradigm can be outlined from our population-level microdiversity analyses (Fig. 5d). As microbes enter the hydrothermal plume, some groups are selected for, and thrive due to substrates provided locally. This promotes the growth of specific populations; meanwhile, constraints in the plume environment cause selection effects and reduce the diversity of the population majority. Higher N/S SNV indicates that these are young populations growing in the plume, with the higher growth rates arising from them consuming primary energy sources such as reduced sulfur compounds. Gene-specific sweeps (and selected genes involving sulfur metabolism) indicate local adaptation to the plume environment and change the genetic structures of populations after migration. Plume microbial populations are still in the early stage of evolution; as time goes on, we predict that mutations will progressively accumulate and deleterious SNVs will be gradually purged.

## Discussion

Sulfur oxidation is the major energy-yielding reaction in hydrothermal plumes. On one hand, it significantly shapes taxonomy, function, and energy landscapes across the three hydrothermal vent sites studied. On the other hand, we observed that distinctive plume geochemistry also influences the energy landscape across the three sites [4, 73]. For instance, other important energy sources, such as methane and hydrogen, also have important roles in the energy landscape of hydrothermal plumes. This highlights the notion of the decisive role of geochemistry on the local energy landscape, especially for plume environments, in which the primary production sources solely come from the substrates entrained in hydrothermal fluids. The existence of a core plume microbiome that was defined in this study indicates that a general biogeochemical feature – energy and substrate supply – within hydrothermal plumes supports the growth of these globally dispersed cosmopolitan microorganisms. As a result, the core plume microbiome is most likely the result of a sulfur oxidation-based energy landscape shared by hydrothermal plumes worldwide. We observed increased taxa abundance and higher growth rates of major energy contributing taxa in plume environments. This supports the interpretation that microbiomes respond to geochemically influenced energy landscapes, with some taxa being fueled by plume substrates.

The above analyses support the theory of an ocean seed bank origin of the hydrothermal plume microbiome [82]. In plume environmental settings, geochemistry defines the substrate and energy availability, serving as a key control on microbiome distribution and abundance [2, 9]. In this scenario, certain microorganisms will be promoted by the environment as a result of the mechanisms of adaptation, and in return, the structure and function of microbial communities are reflections of local environmental conditions. Further, the consistent taxonomy-function-geochemistry links demonstrated by us suggest that omics-based profiling that reflects the entire genetic and functional repertoire of plume microorganisms can be a powerful tool for unraveling the relationship between environment and microbiome.

Characterization of sulfur metabolism in plumes reveals that, while sulfur oxidation is the reaction with the highest MW-score in all plumes, and sulfur-oxidizing genes are highly expressed, the major populations contributing to these processes (*dsrAB* and *sdo* containing MAGs) differ between hydrothermal vent sites. These findings are analogous and similar to observations made by us in another recent study investigating hydrothermal vent chimneys from sites across the world [83]. In these systems, sulfur oxidizing members of Gammaproteobacteria and Campylobacterota were associated with similar ecological guilds and seldom cooccurred, rather their prevalence in a particular site was driven by shifts in geochemistry. Broadly, this demonstrates the variable composition of core sulfur oxidizers in different environments, implying the endemicity of microbial community structure. Core sulfur oxidizers can be derived from the pelagic ocean through stochastic processes that can be influenced by dormancy capacity to provide resilient seed microbes, ocean currents to overcome dispersal limitations, and adaptive strategies to nutrient and temperature fluctuations [2]. Core members of the plume microbiome derived in this manner likely thrive under favorable geochemical conditions [84]. For example, *Pseudomonadales*, *Thiomicrospirales*, and SAR324 are members of the core plume microbiome, but are also known to be abundant cosmopolitan bacteria in the pelagic oceans. These microorganisms can be distributed as seed banks in the global oceans, triggered by plume sulfur substrates, and subsequently become active sulfur oxidizers and thrive in hydrothermal plumes [9, 84].

Sulfur oxidizing microorganisms in the community have metabolic plasticity that allows them to connect with other energy transformation activities, e.g., small carbon substrate utilization, nitrate/nitrite reduction, iron/manganese/arsenite oxidation, and others. This indicates that sulfur and other energy sources can be simultaneously utilized for energy conservation by sulfur oxidizers in various plume environments with different energy landscapes. At the same time, as described in our network complexity analysis, though sulfur oxidation dominates in energy generation, other reactions are also important components in the metabolic network connected to sulfur oxidation, and cohesively contribute to the energy landscape. Sulfur oxidizers mediate the most important energy scavenging reaction of elemental sulfur oxidation as well as other reactions playing a role in energy conservation depending on the local environment; this reflects strategies employed by the plume microbiome for comprehensive utilization of energy sources and adaptation to plume geochemical conditions.

The microdiversity patterns observed in plume microorganisms represent a population selection scheme based on environmental constraints. Low population diversity and high N/S SNV ratio indicate that microbes are selected by plume conditions and actively grow after a short migration history. Evidence shows that gene-specific sweeps within certain plume populations are involved with nutrient uptake, aerobic oxidation, and stress responses, and some sulfur metabolizing genes are also selected during the environmental change. These traits help microbial cells to be more adaptable and resilient in sulfur oxidation-dominated hydrothermal plume conditions. Population alteration in plumes compared to the background deep sea involves both the reshaping of community-level structure and fine-scale strain-level genetic adjustments that include advantageous metabolisms being fixed. These nuanced microdiversity changes can lead to fundamental shifts in population fitness toward niche adaptation. Collectively, the plume microbiome has a distinctive composition, function, and population genetic structure compared to background seawater allowing microorganisms to better adapt to hydrothermal plume conditions. We also demonstrated that plumes exhibit both universal characteristics shared by diverse plumes and specific characteristics unique to each plume. As the environment and associated geochemistry change, the microbiome community and function shift accordingly. The linked relationship between microbiome and biogeochemistry that we demonstrated in this study reflects the overall ecological and evolutionary basis of microbial strategies for thriving in geochemically rich energy landscapes.

## Supplementary information

Supplementary Information and Tables

Supplementary Figures 1-11

Supplementary Data 1

Supplementary Data 2

Supplementary Data 3

Supplementary Data 4

Supplementary Data 5

Supplementary Data 6

Supplementary Data 7

Supplementary Data 8

Supplementary Data 9

Supplementary Data 10

Supplementary Data 11

Supplementary Data 12

Supplementary Data 13

## Data Availability

The MAG genomic sequences are deposited into the NCBI Genome database under the BioProject ID PRJNA488180. The genome annotation results from this study are publicly available at 10.5281/zenodo.5034800 (all plume MAG annotations are deposited to this location).

## Code availability

The Perl and R codes for parsing, calculations, and visualization in this study are publicly available at https://github.com/AnantharamanLab/Hydrothermal_plume_omics_Zhou_et_al._2021.
