# Supplementary material for: Sulfur cycling connects microbiomes and biogeochemistry in deep-sea hydrothermal plumes
Source: ISME J. 2023 May 13;17(8):1194–207. doi: 10.1038/s41396-023-01421-0 (PMC10356823; doi:10.1038/s41396-023-01421-0)
Supplement: Supplementary file 1 — Supplementary Information and Tables [file 41396_2023_1421_MOESM1_ESM.docx]

# Supplementary Information and Tables

## Methods

### Bioenergetic and thermodynamic modeling

To predict the chemical concentrations and activity coefficients in the plume, the equilibrium thermodynamic reaction path modeling approach was adopted. The modeling processes were imposed on the mixtures of seawater and end-member vent fluids from individual hydrothermal environments, including Guaymas Basin, Lau Basin, and Mid-Cayman Rise. The chemical parameters for these seawater and vent fluids samples are provided in Supplementary Table 1. Our thermodynamic modeling builds on the specific plume model implementation of Breier et al[^1^](#_ENREF_1). The estimated temperature of bottom seawater was adopted according to the previous reports[^2^](#_ENREF_2). The original chemical data was derived from Reeves et al[^3^](#_ENREF_3). For each hydrothermal vent system, we choose at least one representative end-member fluid sample(s), respectively (1 for Guaymas Basin, 2 for Mid-Cayman Rise, and 3 for Lau Basin) (Supplementary Table 1). The following is a brief description of how the modeling was conducted in this study, as modified from the detailed description in Anantharaman et al[^4^](#_ENREF_4).

The equilibrium thermodynamic reaction path modeling was based on the mixing process with the ratio of vent fluids vs. seawater as 1: 10,000. This ratio was chosen to resemble the dilution ratio of vent fluids at the non-buoyant plumes in this study. The reaction path modeling was conducted by REACT, a constitutional program implemented in the Geochemist’s Workbench package[^5^](#_ENREF_5). The thermodynamic prediction (both the Gibbs free energy and biomass yields) was calculated by SUPCRT95[^6^](#_ENREF_6) with the temperature range of 1–425°C and the pressure of 500 bar which can cover all known deep-sea hydrothermal vents. The available energy per kilogram plume fluid was estimated by calculating ΔG for the metabolic reactions listed in Supplementary Table 2, using the results of the reaction path model and multiplying ΔG by the concentration of the most limiting reactant. Resulting Gibbs free energy and biomass yields are reported on a per kilogram plume fluid basis.

**Supplementary Table 1. Chemical parameters of hydrothermal vent end-member fluid samples and corresponding bottom seawaters**

| **Hydrothermal vent** | **Guaymas Basin** | | **Mid-Cayman Von Damm**  **(Shallow)** | | **Mid-Cayman Piccard**  **(Deep)** | |
| --- | --- | --- | --- | --- | --- | --- |
| **Properties** | Theme Park | Bottom Seawater | East Summit | Bottom Seawater | Beebe 3 | Bottom Seawater |
| Temp (°C) | 315 | ~2.5 | 226 | 5 | 397 | 5 |
| Mn^2+^ | 0.24 | 0 | 6.50E-14 | 0 | 9.76E-07 | 0 |
| Fe^2+^ | 0.18 | 0 | 4.83E-13 | 0 | 2.07E-03 | 0 |
| Mg^2+^ | 0 | 53.5 | 0 | 52.4 | 0 | 52.4 |
| Cl^-^ | 637 | 538 | 658 | 545 | 358 | 545 |
| pH (at 25°C) | 5.9 | 8 | 5.56 | 8 | 3.17 | 8 |
| H_2_, aqueous | 3.4 | 0 | 19.2 | 0 | 20.7 | 0 |
| H_2_S | 5.98 | 0 | 3.2 | 0 | 12 | 0 |
| ∑CO_2_ | 61.1 | 2.18 | 2.78 | 2.2 | 26 | 2.2 |
| CO | 0 | 0 | 0 | 0 | 0 | 0 |
| CH_4_, aqueous | 63.4 | 0 | 2.84 | 0 | 0.123 | 0 |
| NH_4_^+^ | 13.6 | 0.0001 | 0.0179 | 0.0001 | 0.0347 | 0.0001 |
| O_2_, aqueous | 0 | 0.1^a^ | 0 | 0.25^b^ | 0 | 0.26^c^ |
| NO_3_^-^ | 0 | 0.037^a^ | 0 | 0.018^b^ | 0 | 0.018^c^ |
| NO_2_^-^ | 0 | 0.00003^a^ | 0 | -^b,d^ | 0 | -^c,d^ |
| N_2_^e^, aqueous | 0.48 | 0.58 | 0.48 | 0.58 | 0.48 | 0.58 |

All concentrations are in mmol/kg vent fluid or seawater. *In situ* pH values were calculated by using an equilibrium reaction path model that increased the temperature of the measured fluid to the original vent fluid temperature (*in situ* pH was transformed into the value at 25°C). The estimated temperature of bottom seawater is according to the previous reports[^4^](#_ENREF_4)^,^ [^7^](#_ENREF_7). The original chemical data is derived from the publications of Reeves et al.[^3^](#_ENREF_3) and Anantharaman et al[^2^](#_ENREF_2). For the end-member fluid concentrations of Lau Basin plumes, including Lau Basin Abe (A1 vent), Lau Basin Marine (MA1 vent), and Lau Basin Kilo Moana (KM1 vent), details are provided in Supplementary Table 2 in Anantharaman et al[^2^](#_ENREF_2).

a. Background concentrations at N 20°, W 105° of 1000m (NO_2_^-^) or 2000m (O_2_ and NO_3_^-^) depth in WOCE ATLAS (http://woceatlas.ucsd.edu/) VOLUME2 P18 section.

b. Background concentrations at N 18°, W 67° of 2000m depth in WOCE ATLAS VOLUME3 A22 section. NO_2_^-^ concentration is not applicable.

c. Background concentrations at N 18°, W 67° of 5000m depth in WOCE ATLAS VOLUME3 A22 section. NO_2_^-^ concentration is not applicable.

d. Treated as 0 in thermodynamic modeling.

e. Seawater dissolved N_2_[^8^](#_ENREF_8). Vent fluid dissolved N_2_ concentration is assumed to be 83% of seawater concentration[^9^](#_ENREF_9).

**Supplementary Table 2. Table of metabolic reactions and standard Gibbs free energies at 1, 25, and 100°C.** This table is adapted from the corresponding table from Anantharaman et al[^4^](#_ENREF_4).

| **Metabolism** | **Reaction** |  | **∆G°^a^ (kJ/mol)** | | |
| --- | --- | --- | --- | --- | --- |
|  |  | **e^-b^** | **1°C** | **25°C** | **100°C** |
| H_2_ oxidation (O_2_) | H_2_ + 0.5O_2_ → H_2_O | 2 | -265 | -264 | -260 |
| H_2_ oxidation  (NO_3_^-^ →N_2_) | NO_3_^-^ +2.5H_2_ + H^+^ → 0.5N_2_ + 3H_2_O | 5 | -637 | -637 | -635 |
| Methanotrophy | CH_4_ + 2O_2_ → HCO_3_^-^ + H^+^ + H_2_O | 8 | -828 | -825 | -810 |
| Sulfide oxidation  (O_2_)^c^ | HS^-^ + 2O_2_ → SO_4_^2-^ + H^+^ | 8 | -798 | -793 | -768 |
| Thiosulfate  oxidation (O_2_) | S_2_O_3_^2-^ + 2O_2_ + H_2_O → 2SO_4_^2-^ + 2H^+^ | 8 | -773 | -766 | -736 |
| Thiosulfate  oxidation (NO_3_^-^) | S_2_O_3_^2-^ + 1.6NO_3_^-^ + 0.2H_2_O → 2SO_4_^2-^ + 0.8N_2_ + 0.4H^+^ | 8 | -733 | -728 | -713 |
| Elemental Sulfur  oxidation (O_2_) | S^0^ + 1.5O_2_ + H_2_O → SO_4_^2-^ + 2H^+^ | 6 | -540 | -535 | -512 |
| H_2_ oxidation  (NO_3_^-^ →NO_2_^-^) | NO_3_^-^ +H_2_ → NO_2_^-^ + H_2_O | 2 | -177 | -177 | -175 |
| Elemental Sulfur  oxidation (NO_3_^-^) | S^0^ + 1.2NO_3_^-^ + 0.4H_2_O → SO_4_^2-^ + 0.6N_2_ + 0.8H^+^ | 6 | -510 | -507 | -494 |
| Sulfide oxidation  (NO_3_^-^) | NO_3_^-^ + HS^-^ + H^+^ → NO_2_^-^ + H_2_O + S^0^ | 2 | -170 | -170 | -172 |
| Iron reduction | H_2_ + 2Fe^3+^ → 2 Fe^2+^ + 2H^+^ | 2 | -89.6 | -92.5 | 100 |
| Ammonium  oxidation | NH_4_^+^ + 1.5O_2_ → 2H^+^ + NO_2_^−^ + H_2_O | 6 | -261 | -264 | -271 |
| H_2_ oxidation  (NH_4_^+^ →N_2_) | 0.5N_2_ + 1.5H_2_ + H^+^ → NH_4_^+^ | 3 | -217 | -216 | -207 |
| Sulfate reduction | SO_4_^2-^ + 4H_2_ + H^+^ → HS^-^ + 4H_2_O | 8 | -261 | -264 | -271 |
| Methanogenesis | 4H_2_ + HCO_3_^-^ H^+^ → CH_4_ +3H_2_O | 8 | -232 | -232 | -229 |
| Iron oxidation (O_2_)^c^ | Fe^2+^ + 0.25O_2_ + 2.5H_2_O → Fe(OH)_3,_s + 2H^+^ | 1 | -16.0 | -16.0 | -18.8 |
| Iron oxidation  (NO_3_^-^) | Fe^2+^ + 0.2NO_3_^-^ + 2.4H_2_O → Fe(OH)_3,_s + 0.1N_2_ + 1.8H^+^ | 1 | -11.0 | -11.3 | -15.8 |
| Manganese  oxidation | Mn^2+^ + 0.5O_2_ + H_2_O → MnO_2_,s + 2H^+^ | 2 | -5.65 | -5.71 | -4.62 |

a. Standard Gibbs free energies of reaction predicted by SUPCRT95[^6^](#_ENREF_6).
b. Number of electrons transferred during the reaction.
c. Reactions using particulate and aqueous phase e^-^ donors were predicted individually
(Sulfide and iron oxidation).

**Supplementary Table 3. Membership of the core plume microbiome.** The metabolic characteristics (including capability based on genomes or distribution patterns) were based on currently available reports/publications of the same or sub-microbial groups.

| **Microbial group** | **Phylogeny** | **Metabolic characteristics** | **Potential origin** |
| --- | --- | --- | --- |
| Sva0996 marine group | Actinobacteriota | uncultured marine microorganisms | likely seawater[^10^](#_ENREF_10) |
| *Sulfurimonas* | Epsilonbacteria; Campylobacterales | reduce nitrate, oxidize both sulfur and hydrogen[^11^](#_ENREF_11) | seafloor sediment/subsurface[^12^](#_ENREF_12) |
| SAR202 clade | Chloroflexi | specifically inhabit the aphotic realm; members metabolize multiple organosulfur compounds; many appear to be sulfite-oxidizers[^13^](#_ENREF_13) | seawater[^13^](#_ENREF_13) |
| Marinimicrobia | Marinimicrobia | members contain N_2_O reductase, nitrate reductase, and polysulfide reductase[^14^](#_ENREF_14) | seawater[^14^](#_ENREF_14) |
| JL-ETNP-F27 | Planctomycetota | uncultured marine microorganisms | likely seawater[^15^](#_ENREF_15) |
| Pla3 lineage | Planctomycetota | uncultured marine microorganisms | likely seawater |
| Magnetospiraceae | Alphaproteobacteria; Rhodospirillales | magenetotactic, chemoorganoheterotrophic and chemolithoautotrophic under microaerobic condition (based on *Magnetospira thiophila*)[^16^](#_ENREF_16) | likely seawater[^16^](#_ENREF_16) |
| *Alteromonas* | Gammaproteobacteria; Alteromonadales | chemoorganotrophic, aerobic[^17^](#_ENREF_17) | seawater[^17^](#_ENREF_17) |
|  |  |  |  |
| *Marinobacter* | Gammaproteobacteria; Alteromonadales | utilize a variety of aliphatic and aromatic compounds, both aerobic or anaerobic with nitrate/nitrite[^17^](#_ENREF_17) | seawater[^17^](#_ENREF_17) |
| *Pseudomonas* | Gammaproteobacteria; Pseudomonadales | capable of heterotrophic Mn(II)-oxidation[^18^](#_ENREF_18); members from hydrothermal sediment are potential PAH degraders[^19^](#_ENREF_19) | seawater or marine sediment |
| HOC36 | Gammaproteobacteria | sponge/coral symbiotic microorganisms[^20^](#_ENREF_20)^,^ [^21^](#_ENREF_21) | seawater[^26^](#_ENREF_26)^,^ [^27^](#_ENREF_27) |
| SAR86 clade | Gammaproteobacteria | chemoheterotrophic, aerobic; capable of degrading lipids and polysaccharides[^22^](#_ENREF_22); conducting proteorhodopsin-based photosynthesis[^22^](#_ENREF_22) | seawater[^22^](#_ENREF_22) |
| SUP05 cluster | Gammaproteobacteria; Gammaproteobacteria *incertae sedis* | deep-sea hydrothermal SUP05 cluster can oxidize sulfur and hydrogen[^4^](#_ENREF_4) | seawater[^4^](#_ENREF_4) |
| SAR324 clade | Deltaproteobacteria | degrade aliphatic and aromatic hydrocarbon, and alcohol; oxidize sulfur, methane, and formate[^23^](#_ENREF_23)^,^ [^24^](#_ENREF_24) | seawater[^23^](#_ENREF_23)^,^ [^24^](#_ENREF_24) |

**Supplementary Data and Figures**

**Supplementary Data 1**. Detailed information of hydrothermal plume samples

**Supplementary Data 2**. Sample information for core microbiome analysis and sample characterization map

**Supplementary Data 3**. Genomic properties of MAGs obtained from hydrothermal plume and background

**Supplementary Data 4**. Summary information of functional traits in individual genomes

**Supplementary Data 5**. Microbial group contributions to the metagenome (MetaG) coverage of functional traits. Microbial group contributions to the MetaG coverage of significantly differentiated functional traits (Table 1) within MetaGs from three hydrothermal vent sites. The brown-labeled percentages are the top three in each column. Microbial group contributions to the MetaG coverage of major functions (Table 2) within MetaGs from three hydrothermal vent sites. The brown-labeled percentages are the top three in each column. Bold squares indicate that they are shared between background and plume samples.

**Supplementary Data 6**. Microbial group contributions to the abundance/expression level of functional traits based on metagenome (MetaG) and metatranscriptome (MetaT). Microbial group contributions to the MetaG coverage of significantly differentiated functional traits from Mid-Cayman Von Damm (Shallow) MetaGs (Table 1) (Corresponding to Fig. S8c). The brown labeled percentages indicated major microbial contributors in each column (Only the ones > 30% were labeled). Microbial group contributions to the MetaT expression level calculated by metatranscriptomic reads mapping of significantly differentiated functional traits from Mid-Cayman Piccard (Deep) and Von Damm (Shallow) MetaTs (Table 2) (Corresponding to Fig. S8c). The brown labeled percentages indicated major microbial contributors in each column (Only the ones > 30% were labeled).

**Supplementary Data 7**. DESeq results of MAGs and functional traits between plume and background samples based on metagenome and metatranscriptome datasets from Mid-Cayman hydrothermal environments. DESeq-based statistical analysis of the abundance and gene expression differences of MAGs from Plume vs Background (P-v-B). Positive Log2 Fold Change values were labeled in yellow, while negative Log2 Fold Change values were labeled in light blue.

**Supplementary Data 8**. Thermodynamic modeling for hydrothermal plume environments. Properties and units are listed in the first two rows. The thermodynamic modeling dataset was calculated within the temperature range of 2°C to the end-member vent fluid temperature for each hydrothermal plume environment. Here only thermodynamic modeling results for Mid-Cayman environments were shown. For all results including Mid-Cayman, Guaymas Basin, and Lau Basin environments, refer to the extended data for all (https://doi.org/10.5281/zenodo.5168064).

**Supplementary Data 9**. Energy contribution based on metagenomes. The abundance of each electron donor/acceptor reaction was calculated by adding up the coverage values of all genes that are responsible for the reaction. NO_3_^-^/(O_2_+NO_3_^-^) ratio was used to reflect the oxidative condition for each environment. Energy contribution was calculated by multiplying reaction abundance by the energy yield for each reaction. Reaction and energy yield information refer to Supplementary Table 2.

**Supplementary Data 10**. *Tara* Ocean sample information for inStrain analysis. Only *Tara* Ocean samples with depth ≥ 800 m were used in the inStrain analysis. Metagenomic reads collected from the same station (see Station identifier in the table) were combined as one to represent the microbial community for that station.

**Supplementary Data 11**. inStrain analysis result comparison between hydrothermal environment and *Tara* Ocean datasets. Both hydrothermal environment datasets (either plume or background metagenomic reads altogether) and *Tara* Ocean datasets (only include samples with ≥ 800 m depth; metagenomic reads from one sample station were combined altogether, *Tara* Ocean sample details refer to Supplementary Data 10) were used for mapping. InStrain analyses were conducted and compared within each hydrothermal vent site.

**Supplementary Data 12**. High Fst genes identified from the comparison between hydrothermal environment and Tara Ocean samples. High Fst genes were first identified as these genes have FST value > FST mean + 2.5 × FST std. Then, these genes were further filtered by the following requirements: 1) nucleotide diversity in Hydr < nucleotide diversity genome average in Hydr; 2) N/S SNV ratio in Hydr > N/S SNV ratio genome average in Hydr; 3) nucleotide diversity in Hydr < nucleotide diversity in Tara; 4) N/S SNV ratio in Hydr > N/S SNV ratio in Tara. "Worksheet1" shows the parameters of all identified high FST genes. "Worksheet2" shows the annotation result of all identified high FST genes. Abbreviations: "Hydr" stands for hydrothermal environment, "Tara" stands for Tara Ocean samples, "N/S SNV ratio" stands for nonsynonymous (N) to synonymous (S) SNV ratio.

**Supplementary Data 13**. Evolutionary analysis results of sulfur metabolizing genes based on the comparison between the hydrothermal environment and Tara Ocean samples. The FST, nucleotide diversity, and N/S SNV ratio of sulfur metabolizing genes (including sat, aprA, sdo, dsrAB, and soxBCY) were calculated for individual genomes. The signal of being fixed after migration (Hydrothermal environment vs Tara Ocean) was obtained by meeting the four criteria: 1) FST > FST mean, 2) nucleotide diversity in Hydr < that in Tara, 3) N/S SNV ratio in Hydr > that in Tara, and 4) Both coverages in Hydr and Tara ≥ 5. These criteria were relatively less stringent compared to those in Supplementary Data 12 ("Worksheet1"), while they still suggested positive gene fixation signals.

**Supplementary Figure S1**. Schematic diagram of hydrothermal vent structure and sampling positions. (a) Schematic diagram indicating detailed sample positions. (b) Summary table of DNA and cDNA sequencing libraries within this study.

**Supplementary Figure S2**. Compositional bar plot of the hydrothermal plume and background microbial community at the phylum level. Microbial community analysis was based on 47 hydrothermal plume and background 16S rRNA gene datasets.

**Supplementary Figure S3**. PCoA diagrams of the global hydrothermal plume and background microbial community based on 16S rRNA gene. Four subpanels contained PCoA diagrams each based on weighted/unweighted Unifrac distance (a, b based on weighted Unifrac distance; c, d based on unweighted Unifrac distance) and labeled by location/sample characteristics (plume or background) (a, c labeled by sample characteristics; b, d labeled by location characteristics).

**Supplementary Figure S4**. Phylogenetic tree of MAGs based on concatenated 16 ribosomal proteins. Only the nodes with ultrafast bootstrap (UFBoot) support values over 90% were labeled with black dots. The functional traits for each MAG were parsed and labeled in the tree according to the HMM scan result. Filled shapes indicate presence of functional traits, blank shapes indicate absence of functional traits.

**Supplementary Figure S5**. Phylogenetic tree based on 16S rRNA gene from each MAG. The bootstrap (UFBoot) support values were labeled to each node (only showing those > 80%). Taxonomic labels were according to both SILVA_128_SSUParc_tax_silva database BLAST result and MAG 16RP phylogenetic tree.

**Supplementary Figure S6**. Phylogenetic tree of concatenated *dsrAB* encoding proteins and gene structure figure of *dsr* containing scaffolds. (a) Phylogenetic tree of concatenated *dsrAB* encoding proteins. DsrA and DsrB were aligned with reference sequences independently and concatenated. The concatenated protein alignment was trimmed with gap threshold of 25% using trimAl v1.2. The phylogenetic tree was reconstructed by IQ-TREE v1.6.9 with settings as described in the methods. UFBoot bootstrap values were labeled at each node. Genomes from this study were highlighted in yellow. (b) Gene structure figure of *dsr* containing scaffolds. The assignment of each *dsr* gene component was confirmed by combining BLAST result in NCBI database and position in the *dsr* gene operon. This figure was visualized in Geneious Prime v2020.2.3.

**Supplementary Figure S7**. Sunburst figures and tables representing the comparison of MAG taxonomic composition and abundance and functional trait of samples from three hydrothermal environments based on metagenomic read mapping results. (a) sunburst figures and table for comparison of MAG taxonomic composition. The mean MetaG MAG coverage values of background (B) and plume (P) were used to draw the sunburst figure. DESeq-based statistical analysis on the abundance difference of MAGs that are affiliated to certain microbial groups indicated significantly differentiated microbial groups between the comparisons of each two out of three environments. Only the microbial groups with > 5% mean coverage percentage in either one of the environments were listed with the corresponding Log2 Fold Change values and adjusted *p* values (by nbinomWaldTest). (b) Table for comparison of functional traits. DESeq-based statistical analysis on the abundance of functional traits indicated significantly differentiated functional traits between the comparisons of each two out of three environments. The corresponding Log2Fold Change values and adjusted *p* values (by nbinomWaldTest) were labeled for each identified functional trait.

**Supplementary Figure S8**. Sunburst diagrams and heatmaps representing DESeq result of MAG and functional traits between plume and background samples based on metagenome and metatranscriptome datasets from Mid-Cayman hydrothermal environments. (a) Sunburst diagrams of MAGs based on metagenome and metatranscriptome. DESeq-based statistical analysis on the abundance and active abundance difference of MAGs from P-v-B (Plume vs Background) comparisons indicated MAGs with significant adjusted *p* values (*p* < 0.05, red or blue star labeled) have differentiated abundances/active abundances in different hydrothermal eco-niches. Red stars indicated positive Log2 Fold Change, while blue stars indicated negative Log2 Fold Change; Log2 Fold Change values were also labeled with the stars accordingly. Only microbial groups with > 3% relative abundance in sunburst diagrams were labeled, while minor microbial groups were grey-colored. (b) Heatmap indicating MAG abundance difference based on metagenome and metatranscriptome. (c) Heatmap indicating functional trait abundance difference based on metagenome and metatranscriptome. Corresponding abundances shown here were of significant adjusted *p* values (by nbinomWaldTest). Log2 Fold Change values were labeled accordingly. Relative abundance at row was normalized by removing the mean (centering) and dividing by the standard deviation (scaling). Resulted Row Z-score bars were presented accordingly in individual subpanels.

**Supplementary Figure S9**. Thermodynamic estimation of available free energies and biomasses from reactions of electron donors in various hydrothermal plumes. The estimated biomasses and free energies of individual environments were normalized to percentage fractions. Dotted lines (one at 2.95℃ and one at 4.9℃) showed two temperatures that we picked to conduct the biomass and free energy estimations for representing upper and lower plume temperatures. The abbreviation of reaction was labeled as: “S:O_2_” standing for the reaction of sulfur as the electron donor and oxygen as the electron acceptor.

**Supplementary Figure S10**. MW-scores (metabolic weight scores) and functional network diagrams of three hydrothermal vent sites. (a) MW-score table and functional network diagram of Guaymas Basin. (b) MW-score table and functional network diagram of Mid-Cayman Rise. (c) MW-score table and functional network diagram of Lau Basin. MW-score tables and functional networking diagrams were generated by METABOLIC-C software using plume metagenomic reads as inputs. MW-score reflects abundance fraction of individual metabolic steps within the whole community. In each MW-score table, MW-scores are given in a separated column, and the rest part of the table indicates the contribution percentage to each MW-score of the genomes within the community as grouped by each phylum. From METABOLIC-C standard MW-score results, a group of metabolic cycling steps that are important on reflecting the plume substrate metabolisms were selected to make functional network diagrams. In each diagram, the size of a node is proportional to the gene coverage associated with the metabolic/biogeochemical cycling step. The thickness of the edge was depicted according to the average of gene coverage values of the two connected biogeochemical cycling steps. The color of the edges was assigned based on the taxonomy of the represented genome.

**Supplementary Figure S11**. Plume environment networks and network complexity diagram. (a, b) Plume environment networks based on reactions. The reactions and energy yields for each reaction were based on thermodynamic estimation results at two representative temperatures in plume environments, 3.0°C (a) and 4.9°C (b). In each network, substrates and products (left side) were connected to each reaction (right side) by an arrow with direction. The size of each reaction was proportional to its energy yield. (c) Network complexity diagram representing each reaction’s influence on the complexity of the network. In the figure, different colors stand for different hydrothermal environments, different symbol shapes stand for different reactions. The substrates (including electron donor and acceptor) are listed for each reaction in the legend. The x-axis is the change in complexity (ΔC) of the whole network for a node (a rection here) and the y-axis is the percent energy yield of that reaction in the whole community. This network complexity diagram was based on thermodynamic estimation results at 4.9°C.

**References**

1. Breier J*, et al.* Sulfur, sulfides, oxides and organic matter aggregated in submarine hydrothermal plumes at 9°50'N East Pacific Rise. *Geochim Cosmochim Acta* **88**, 216-236 (2012).

2. Anantharaman K, Breier JA, Dick GJ. Metagenomic resolution of microbial functions in deep-sea hydrothermal plumes across the Eastern Lau Spreading Center. *ISME J* **10**, 225 (2015).

3. Reeves EP, McDermott JM, Seewald JS. The origin of methanethiol in midocean ridge hydrothermal fluids. *Proc Natl Acad Sci U S A* **111**, 5474 (2014).

4. Anantharaman K, Breier JA, Sheik CS, Dick GJ. Evidence for hydrogen oxidation and metabolic plasticity in widespread deep-sea sulfur-oxidizing bacteria. *Proc Natl Acad Sci U S A* **110**, 330 (2013).

5. Bethke CM. *Geochemical and biogeochemical reaction modeling*. Cambridge University Press (2007).

6. Johnson JW, Oelkers EH, Helgeson HC. SUPCRT92: A software package for calculating the standard molal thermodynamic properties of minerals, gases, aqueous species, and reactions from 1 to 5000 bar and 0 to 1000 °C *Computers & Geosciences* **18**, 899-947 (1992).

7. Anantharaman K, Duhaime MB, Breier JA, Wendt K, Toner BM, Dick GJ. Sulfur Oxidation Genes in Diverse Deep-Sea Viruses. *Science* **344**, 757-760 (2014).

8. Weiss R, Craig H. Precise shipboard determination of dissolved nitrogen, oxygen, argon, and total inorganic carbon by gas chromatography. In: *Deep Sea Research and Oceanographic Abstracts*). Elsevier (1973).

9. Brandes JA, Boctor NZ, Cody GD, Cooper BA, Hazen RM, Yoder Jr HS. Abiotic nitrogen reduction on the early Earth. *Nature* **395**, 365 (1998).

10. Li J*, et al.* Characterization of particle-associated and free-living bacterial and archaeal communities along the water columns of the South China Sea. *Biogeosciences* **18**, 113-133 (2021).

11. Han Y, Perner M. The globally widespread genus *Sulfurimonas*: versatile energy metabolisms and adaptations to redox clines. *Front Microbio* **6**, 989-989 (2015).

12. Dick GJ. The microbiomes of deep-sea hydrothermal vents: distributed globally, shaped locally. *Nat Rev Microbiol* **17**, 271-283 (2019).

13. Mehrshad M, Rodriguez-Valera F, Amoozegar MA, López-García P, Ghai R. The enigmatic SAR202 cluster up close: shedding light on a globally distributed dark ocean lineage involved in sulfur cycling. *ISME J* **12**, 655-668 (2018).

14. Hawley AK*, et al.* Diverse Marinimicrobia bacteria may mediate coupled biogeochemical cycles along eco-thermodynamic gradients. *Nat Commun* **8**, 1507 (2017).

15. Fernandes GL, Shenoy BD, Damare SR. Diversity of Bacterial Community in the Oxygen Minimum Zones of Arabian Sea and Bay of Bengal as Deduced by Illumina Sequencing. *Front Microbio* **10**, 3153 (2020).

16. Williams TJ, Lefèvre CT, Zhao W, Beveridge TJ, Bazylinski DA. *Magnetospira thiophila* gen. nov., sp. nov., a marine magnetotactic bacterium that represents a novel lineage within the Rhodospirillaceae (Alphaproteobacteria). *Int J Syst Evol Microbiol* **62**, 2443-2450 (2012).

17. Rosenberg E, DeLong EF, Lory S, Stackebrandt E, Thompson F. *The Prokaryotes: Gammaproteobacteria*. Springer Berlin Heidelberg (2014).

18. Dick GJ, Tebo BM. Microbial diversity and biogeochemistry of the Guaymas Basin deep-sea hydrothermal plume. *Environ Microbiol* **12**, 1334-1347 (2010).

19. Wang W, Li Z, Zeng L, Dong C, Shao Z. The oxidation of hydrocarbons by diverse heterotrophic and mixotrophic bacteria that inhabit deep-sea hydrothermal ecosystems. *ISME J* **14**, 1994-2006 (2020).

20. Cleary DFR, Polónia ARM, Reijnen BT, Berumen ML, de Voogd NJ. Prokaryote Communities Inhabiting Endemic and Newly Discovered Sponges and Octocorals from the Red Sea. *Microb Ecol* **80**, 103-119 (2020).

21. Apprill A, Weber LG, Santoro AE. Distinguishing between Microbial Habitats Unravels Ecological Complexity in Coral Microbiomes. *mSystems* **1**, e00143-00116 (2016).

22. Dupont CL*, et al.* Genomic insights to SAR86, an abundant and uncultivated marine bacterial lineage. *ISME J* **6**, 1186 (2012).

23. Sheik CS, Jain S, Dick GJ. Metabolic flexibility of enigmatic SAR324 revealed through metagenomics and metatranscriptomics. *Environ Microbiol* **16**, 304-317 (2013).

24. Cao H*, et al.* Delta-proteobacterial SAR324 group in hydrothermal plumes on the South Mid-Atlantic Ridge. *Sci Rep* **6**, 22842 (2016).
